# Implementation of Brain Breaks^®^ in the Classroom and Effects on Attitudes toward Physical Activity in a Macedonian School Setting

**DOI:** 10.3390/ijerph15061127

**Published:** 2018-05-31

**Authors:** Biljana Popeska, Snezana Jovanova-Mitkovska, Ming-Kai Chin, Christopher R. Edginton, Magdalena Mo Ching Mok, Serjoza Gontarev

**Affiliations:** 1 Faculty of Educational Sciences, Goce Delcev University, Krste Misirkov 10–A, Stip 2000, Macedonia; snezana.jovanova@ugd.edu.mk; 2 HOPSports, Inc., 4262 Blue Diamond Road #102-359, Las Vegas, NV 89139, USA; mingkai@hopsports.com; 3 Human Performance Center, University of Northern Iowa, 105, Cedar Falls, IA 50614, USA; christopher.edginton@uni.edu; 4 Department of Psychology and Assessment Research Centre, The Education University of Hong Kong, 10 Lo Ping Road, Tai Pol, Hong Kong, China; mmcmok@friends.eduhk.hk; 5 Faculty for Physical Education, Sport and Health, University “St. Chyril and Methodius”, Zeleznicka bb, Skopje 1000, Macedonia; gontarevserjoza@gmail.com

**Keywords:** video exercises, primary school children, physical activity, attitudes, Brain Breaks^®^

## Abstract

The purpose of this study was to explore the effects of Brain Break^®^ activities on interest and motivation for physical activity among schoolchildren and the contribution of such activities on learning for health and holistic development. The study sample was comprised of 283 participants, primary school students from 3rd to 5th grades from two public schools in the Republic of Macedonia. Six experimental and six control groups were included in the study. Interventions in classroom settings—based Brain Break^®^ video exercises were introduced in the experimental group during a period of three months. Students’ attitudes toward physical activity were tested using a self-report survey instrument entitled “Attitudes toward Physical Activity Scale (APAS)” before and after intervention. Applied factor analyses were completed and the results of these analysis support APAS validity and the successful use of this application in the measurement of the learning experience, self-awareness, self-efficacy, and self-confidence in developing physical fitness. Learning was enhanced by using video exercises. Information presented in this paper is meaningful for the promotion of better exercise habits and the holistic approach to better health by using personal motivation and motivation provided by others. The results from repeated ANCOVA suggest positive effects of the applied Brain Break^®^ video exercises as an interventional program. The study confirms the effect of application of Brain Break^®^ video exercises on children’s attitudes for physical activity, motivation for PA, internalization of movement habits as personal good.

## 1. Introduction

Living in a world empowered by technology, we cannot disregard the use of the media’s influence on education and human development. This influence could be both positive and negative. Physical activity has huge impact on children’s growth and development. Despite the increased numbers of studies that emphasize the positive effects of physical activity in children [1,2,3,4,5], a large number of studies report on the benefits from regular physical activity and greater opportunities for involvement of children in different forms of physical activity and sports. Unfortunately, a growing number of studies show a trend of decreasing physical activity level among children, while an increase in health problems is reported for children [6,7,8,9]. Poor eating habits, modern technology and computerization are some of the factors that are frequently identified as the reasons for physical inactivity in children [10,11,12,13,14]. Nearly all of them are related to contemporary ways of living and living conditions. Their effect is equally present both in school and in home surrounding. 

As a part of modern life, technology is composed part of children’s’ lifestyle. Children born in the first part of this millennium are identified as the “iGeneration” [15]. Speaking about modern way of living and use of technology, sedentary behavior usually assessed as screen time and predominantly TV viewing is found associated with unhealthy daily behavior among children and adolescents [10,11,14,16,17,18]. As result of this, most of the children fail to meet basic physical activity recommendations [19]. Increasingly technological sedentary behaviors are also associated with TV/DVD video viewing, using the computer for non-homework purposes, playing video games etc. [10,20]. However, technology could have both bad and good effects on physical activity level of children and their active lifestyle. In this regard, many studies also reported that technology could be effectively used in promotion of active, healthy lifestyles in society [21] including schools as well [22,23,24]. Interactive video games and internet-based physical activity interventions have become more attractive to stimulate children’s interest and to get them engaged more in active movements [25]. 

When referring to physical activity in schools, the most frequent excuses to neglect physical activity occurs because of a crowded curriculum with more time allocated for the adoption of cognitive knowledge, extensive homework, and numerous extracurricular activities of children that are generally performed in a sitting position [18]. However, the success of education excellence for Finnish children has created a challenge for frequent testing and large amount of time in studying but to the increase in student learning and in daily physical activity contributes to better physical and cognitive development [26,27,28]. 

When speaking about physical activity level of children in school, always the first association is related with physical education classes. With decades, schools are called to promote and provide physical activity. Physical education is strongly recommended as essential access point to provide and promote physical activity for all children, and as the only venue where the least active children experience physical activity at higher intensities [29]. However, not always schools and physical education are able to answer on this. Many problems in physical education process in schools are present. Most of the problems are related with the lack of sport infrastructure, increased class sizes, lack of equipment resulting with a lot of ineffective time [30,31], budgetary reductions and a curriculum that does not rely on the real interests of children [29,30,31,32]. The reduction of time allocated for physical education programs, increased class sizes, budgetary reductions, and other contributing factors have led to dismissal of physical education and the provision of facilities in support of such programs [29,32]. Referring to school conditions in the Republic of Macedonia, limited material and space facilities for physical education classes has been noted [30,33]. Current physical and health education curriculum does not rely on childrens’ interests and although is designed to support holistic development and integrates health aspects, during the process of realization, these elements are missing. There is an evident lack of extracurricular programs aimed to promote healthy and active lifestyle, as well as lack of programs that use technology as manner of motivation for physical activity

Some of the above-mentioned aspects and problems suggest that intervention is necessary in direction of finding a form within the framework of regular classes which will increase the level of physical activity of children while they are at school. Considering the amount of time that children spent in school, school environment is recognized as ideal for implementing different physical activity interventions [3]. This also includes the classroom as a place for physical activity of children. One of those forms that allow increasing the level of physical activity in children that can be applied in the classroom is the technology supported Brain Break^®^ platform. 

As for the effects of classroom based physical activities, findings from numerous studies emphasize their positive impact on increasing the level of physical activity among children [23,34,35], positive effect that physical activity and active break have on cognitive functions and brain health [36,37,38,39]. Different studies have also reported that following a bout of physical activity, children exhibit enhancements in attention and on-task behaviors [40,41]. The benefits from classroom based physical activities implemented in the context of Macedonian schools could also be noted from the point of effects from environmental conditions. Namely, in the last decade, the Republic of Macedonia faces a huge problem of air pollution in the winter period. Levels of air pollution in the Republic of Macedonia are among the highest in Europe [42] and research studies have reported a negative relationship of air pollution and pulmonary health, including children who are more sensitive to air pollution than adults [43,44,45]. Sunyer et al. [46] reported that children of 39 schools in Barcelona (Catalonia, Spain) affected by higher traffic-related air pollution showed lesser enhancement in cognitive development. Similarly, in the past 10 years, the atmospheric pollution levels are increased to a toxic level to humans in North China including Beijing, the Yangtze River Delta, and the Pearl River Delta [47,48]. Outdoor sports activities for primary and middle schools were ordered to stop in exceedingly polluted districts in order to preserve children’s health [49,50]. The results from a study conducted by Sichletidis et al. [51] realized on a sample of 3.559 children aged 9–12 suggest that environmental pollution has a detrimental effect on children’s respiratory system. The highest values of rhinitis and infectious bronchitis were noted in children who live in highly polluted regions. In the case of the Republic of Macedonia, Macedonian air quality assessment report for the period 2005–2015 (2017) [52] analyzes different aspects of air pollution including its negative impact on health and life quality. Negative health impacts including lower quality of life, decrease in working ability and premature death are estimated to be huge economic costs affecting all Macedonian citizens [53]. In cases of low air quality, classroom-based physical activity such as doing Brain Break^®^ is a good alternative to outdoor physical activity when the Air Quality Index is unfavorable.

Regarding the positive effects of the implementation of technology as a motivation for physical activity and recommendations from Global Forum GoFPEP 2016 indicate, “Technology is greatly influencing pedagogical strategies. It can serve to complement the efforts of the physical education teacher as a tool to improve engagement and in the assessment process by assisting in the learning, performance and motivational processes. Certainly, technology can assist in recording performance and results. There should be a balance between the use of technology for teaching purposes and assessment in physical education classes in school settings” [54] (p. 38). In this regard, this study presents the implementation of classroom based and technology supported Brain Breaks^®^ Physical Activity Solutions, applied with primary school children in Macedonia.

### Purpose of Research

The purpose of this study was to explore the effects of Brain Break^®^ activities on the interest and motivation for physical activity among schoolchildren and the contribution of such activities to learning about health and holistic development. The main hypothesis is that active participation in classroom-based, physically active breaks on regular daily bases will have positive effects on the level of physical fitness, self-efficacy, goals orientation, interest for physical activity, self-awareness of the importance and benefits of physical activity and its contribution to learning about health and holistic development.

## 2. Methodology

### 2.1. Study Design and Participnts

The study adopted an experimental design involving two groups, control group with no intervention and experimental group for which the intervention-technology supported Brain Break^®^ Solutions were introduced.

The study sample was comprised of 283 primary school students from two public schools in two different communities in Republic of Macedonia, Stip and Strumica. Students from the 3rd, 4th, and 5th grades were used as subjects in this study. From each school, two different classrooms for each grade level were assigned either as an experimental classroom or as a control classroom. The study adopted an experimental design where experimental factor-technology supported Brain Break activities were introduced in the experimental group. From the total sample of participants, 152 (54%) were in the experimental group and the other 131 (46%) were participants in the control group. Regarding the gender, 155 (55%) were males and 128 (45%) were females. The sampling distribution by grades and gender is presented in Table 1. The participants were recruited by convenience sampling. Ethics approval was obtained from the Ethical Board at Goce Delcev University in Stip, Macedonia and from the school review board of both schools included in the study. Written and oral informed consents were obtained from both parents and children in groups. 

### 2.2. Instruments

The study used a self-report survey instrument entitled “Attitudes toward Physical Activity Scale (APAS)” that was developed and validated by Mok et al. [55]. The instrument was designed to investigate the effects of providing Brain Break^®^ video exercise lessons to primary school children relating students’ attitudes, beliefs and self-efficacy toward physical activity. The primary version of APAS was designed as a part of a large project applied worldwide in several countries including Lithuania, Poland, Serbia, Turkey, Romania, Croatia, and South Africa [55]. Beside demographic section, APAS questionnaire consists seven sections composed from different number of items related with several aspects of engagement in physical activity. They are following: (1) Promoting the holistic health: a 10-item scale constructed to measure students’ attitudes toward the effectiveness of physical activities in promoting holistic health; (2) Importance of exercise habit: a 5-item scale that measure students’ attitude toward the importance of physical activity as a lifestyle; (3) Self-efficacy in learning with video exercises: a 11-item scale constructed to measure students’ self-efficacy in learning different curriculum subjects by using video exercises; (4) Self-efficacy in using video exercises: 4-item scale designed to measure students’ self-confidence to administrate video exercises independently by themselves; (5) Exercise motivation and enjoyment: a 15-item scale designed to measure students’ motivation and enjoyments to do physical exercise; (6) Self-confidence on physical fitness: an 8-item scale designed to measure students’ self-perception of physical fitness; (6) Trying to do personal best: a 5-item scale designed to measure students’ personal goal orientation and achievements in physical activity.

Participants were invited to respond to each item of the above-mentioned seven sections using a four-point Likert response scale with response categories: Strongly Disagree, Disagree, Agree, and Strongly Agree. 

The questionnaire applied in this study was the original English version translated into Macedonian, the native language of the participants in the study with forward-and back-translation. Translated in this way, the questionnaire was adapted in order to ensure conceptual equivalence to the original one. Some cultural adaptations were made as well. The process of translation was done by a team of experts comprising university professors in Physical Education, Pedagogy, English language and primary school teachers. The questionnaire was completed by the students themselves. Pre-test was made before the implementation of technology supported Brain Break^®^ activities. Post-test was done three months later, after the experimental period was finished. Pre-test and post-test were made in the same period for both the experimental and the control groups in both schools. The original English version of APAS has been validated in previous studies using Rasch analyses [55]. In our study, the internal consistency was established within Cronbach’s alpha test ranged from 0.91 to 0.71. 

### 2.3. Procedures

Following the positive experiences and results from the implementation of Brain Break^®^ in Croatia, Turkey and several other countries [24,35,55], the initiative for the implementation of technology supported Brain Break^®^ Physical Activity Solutions in primary schools in Macedonia started in 2015. The implementation was realized in three phases: (1) preparation phase—familiarization with the concept of technology supported Brain Break^®^ video exercises and partnership agreements with schools; (2) experimental phase—implementation of Brain Break^®^ video exercises solutions, and (3) final phase—data collection and analyses. During the preparation phase, the concept of technology supported Brain Break^®^ video exercises was presented to teachers and principals in several primary schools in Macedonia. Emphasis was given on the technical requirements, the protocols of using Brain Break^®^ video exercises in the classroom, the possibilities for cross subject and cross-curricular learning, holistic approach, and the adaptation requirements of the national curriculum. The final selection of school and classes included in the experiment was done based on the fulfilled technical requirements and the interest of teachers for participation in the study. Technical requirements such as: lap top or PC in the classroom, LCD projector, good and constant internet connection and fast internet were mandatory in each experimental class during the experiment phase. Interest for participation in the experiment demonstrated by the teachers, was another criteria for selection of experimental classes and one of the key factor for successful realization of the experiment. Two research assistants instructed teachers from experimental groups on how to implement the intervention and how to administrate pre-test and post-test. Instructions for teachers pointed the use of Brain Break^®^ video exercises during the day, manners of selection of the video exercises and how to make notifications of students’ feedback. Each teacher received an individual access to the platform. Online access to the official project website: http://hopsports.com/ brain-breaks was available at all times. Control groups were selected based on two criteria: average success of students in particular grade and approximately equal number of pupils compared with prior selected experimental groups. 

The intervention with technology supported Brain Break^®^ video was carried out in two elementary schools in two different cities in Macedonia. The structure of the experimental and control group was equal in both schools. In particular, one 3rd grade, one 4th grade and one 5th grade participated in the experimental group in one school and equally the same structure was maintained in the control group in the same school. The intervention in the experimental group was done in a period of three months, from March to May, 2015. It included implementation of 3–5 min classroom based and technology supported Brain Break^®^ video exercises each day during the period of three months. The active breaks were applied each school day, five days per week during one particular class selected by the teacher. During the period of implementation, teachers from experimental classes involved in the study used digital platform developed and provided by HOPSports^®^ [56]. It is an interactive platform with an innovative physical activity program for schools with free on-line access for teachers. Different videos that integrated basic movements, sport elements, dance and games, performed by animated and real-life instructors, are provided in each Brain Break^®^ video. Furthermore, the contents of videos include knowledge and training for healthy living, nutrition, environment protection as well as mathematics, language and writing skills, art, music and cultural knowledge. 

The selection of the videos, selection of the class, time allocation during the class and period of the day during which Brain Break^®^ activities should be performed were left to be a free choice of the teachers and students. The only requirement was not to use the same video consecutively three times. During this period of three months, the control group had no contact with Brain Break^®^ activities. Pre-test and post-test of both the experimental and the control groups were carried out before and after the intervention using an adapted Macedonian version of APAS. 

### 2.4. Statistical Analyses

The data were analyzed using the statistical package SPSS 22.0 software for Windows (SPSS Inc., Chicago, IL, USA). Basic descriptive statistics were computed. Data normality distribution was determined using skeweness and kurtosis. Crombach alpha coefficient was calculated to determine the internal consistency of the instrument. The factor structure of the Macedonian version of APAS was determined using exploratory factor analyses. Sampling adequacy for factor analysis was determined using the Kaiser-Meyer-Olkin (KMO) test. The factor structure was obtained using the Principal component analyses, Gutman-Kaiser criteria and Varimax rotation in SPSS. The following criteria were used to determine common factors underpinning the items: (1) The loading of the item on the factor >0.40; (2) at least three items with factor loading >0.40 to compose one factor, and (3) items should not cross-load on the other factors with a loading >0.30. The effects of applied video exercises intervention on APAS scores was analyzed using the repeated measures analyses of covariance (ANCOVA) with Time as the within-subject factor and Group (experimental vs control) as the between-subject factor. Gender and age were included in the ANCOVA as covariates. The partial eta-squared (ή^2^) effect sizes for the tests were calculated to indicate the magnitude of the effect. 

## 3. Results

The analyses of data show evidence in support of homogeneity of variance of both the experimental and the control groups. The values of skewness and kurtosis indicated normal distribution of the variables. 

Exploratory factor analysis of data on the Macedonian version of the Attitudes toward Physical Activity Scale (APAS) was conducted using principal component analyses. Initially, 57 items were analyzed. The following two items: (2d) “Being physically active is something I would not give up in my life” and (5m) “I think other children enjoy doing physical activity”, failed to match the a priori listed criteria and they were deleted from subsequent analyses. In addition, five items had loadings >0.40 for two different factors. They were included in the factor in which they have higher load. These items were: (1j) “Being physically active helps to improve my school work”; (5i) “I think better after physical activity”; (1a) “Being physically active helps to make me fit”; (1d) “Being physically active helps to improve my analytic skills”; and (4a) “I know how to choose physical activity in video exercise that suits me”. 

Exploratory factor analysis of the 55 items yielded adequate matrices indices (Bartlett’s test of Sphericity of χ^2^ (1596) = 9818.920 (*p* < 0.01) and Kaiser-Meyer-Olkin measure of sampling adequacy). Principal component analyses followed by the Varimax rotation were conducted, and using the Kaiser-Guttman “eigenvalues greater than one” criterion [57,58,59] the analysis identified seven meaningful factors with eigenvalues ranging from 16.17 to 1.38 and the factor loadings ranging from 0.411 to 0.785. The final factor structure defined by these seven factors accounted for 55.98% of the total variance (Table 2). The values of Cronbach’s alpha test are ranged from 0.91 to 0.74. (0.91 for Self-efficacy in learning with video exercises; 0.90 for Self-confidence on physical fitness; 0.89 for Enjoyment and exercise motivation; 0.81 for Importance of exercise habit for health; 0.74 for Promoting the holistic health and 0.74 for knowledge and self-awareness for individual application of BB video). Obtained values suggest on very high internal consistency for five isolated factors and acceptable for two of the isolated factors. 

Eleven items designed to measure learning experience of children using Brain Break^®^ video activities as well as the effect of Brain Break^®^ videos in holistic personal development and cross-subject relations had the highest projection and saturation on the first isolated factor, named Self-efficacy in learning with video exercises (F1). Example items of this factor are, “I learned about culture through video exercise,” and “I learned about language through video exercise”. 

Eight items designed to measure children’s self-perception of different aspects of physical fitness represented the second isolated factor, named Self-confidence in physical fitness (F2). Example items are, “I am confident with my hand-eye coordination,” and “I am confident with my agility”. 

Twelve items designed to measure the motivation and enjoyment of the participants to be physically active loaded strongly on the third factor, named Exercise motivation and enjoyment (F3). Example items are, “I think my classmates enjoy doing physical activity,” and “I feel better after physical activity.” 

Seven items designed to measure the awareness for creating an exercise habit as well as the effects of movement on personal health loaded on the fourth factor, named Importance of exercise habit for health (F4). Example items are, “It is important to form a habit of being physically active,” and “Being physically active helps to give me good health”.

Six items designed to measure personal best goal orientation of the participants and motivating others to engage as well loaded strongly on the fifth factor, named Training for personal best and motivating others (F5). Example items are, “I seek to explore my best potential in physical activity,” and “I persuade my friends to join me in doing physical activity”. 

Six items designed to measure participants’ attitudes toward effectiveness of technology support Brain Break^®^ activities in promoting holistic health, particularly its effects on cognitive and conative aspect loaded strongly on the sixth factor, named Promoting holistic health (F6). Example items are, “Being physically active helps to reduce my anxiety,” and “Being physically active helps to enhance my self-concept”.

Four items designed to measure the attitudes of participants to their personal knowledge and preparedness for individual independent application of Brain Break^®^ videos loaded strongly on the seventh factor, named Knowledge and self-awareness of individual application of BRAIN BREAK video (F7). Example items are, “I know how to do physical activity if there is a video exercise to follow,” and “I know how to choose physical activity in video exercise that suits me”.

Pearson Product Moment Correlation coefficients among the seven factors identified from the factor analysis are presented in Table 3. The correlation coefficients ranged from 0.209 (between F1 Self-efficacy in learning with video exercises and F2 Self-confidence on physical fitness) and 0.611 (between F3 Exercise motivation and enjoyment and F5 Training for personal best and motivating others) and were all statistically significant (*p* < 0.01).

Table 4 presents the pre-test and post-test mean scores for the experimental and the control groups, as well as the effect sizes (ή^2^ and partial ή^2^) of the differences. If we analyze the mean values of the experimental and the control groups at pre-test, differences between the values of the two groups could be noted, especially for the F6 Promoting holistic health and F7 Knowledge and self-awareness for individual application of Brain Break^®^ video. 

**Figure 1:**
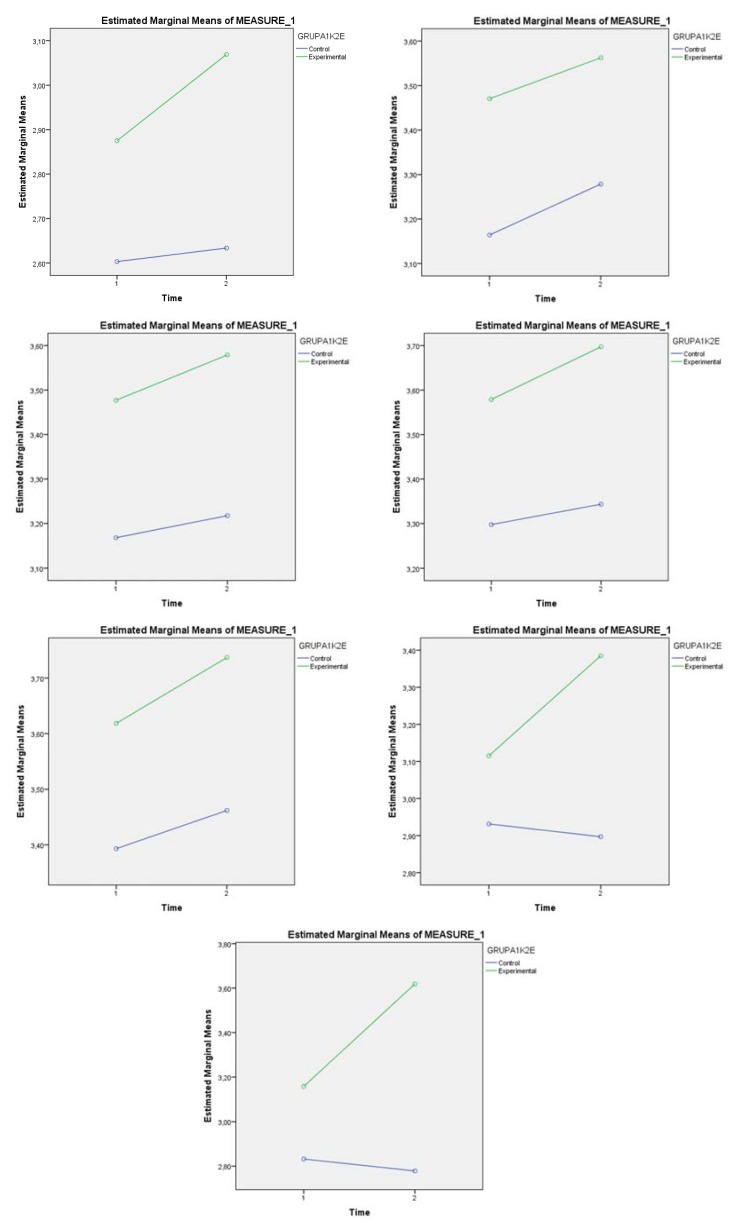
Scale values of the experimental and the control groups at pre-test and post-test.

After the intervention and effects of the experimental factor-implementation of Brain Break video exercises, the differences in means between the experimental and the control groups at post-test are statistically significant. These differences all showed greater effects in the experimental group because of the impact of the experimental intervention. Obtained indicators for Partial ή^2^ (Time) according the analyses of Cohen (1988) [60], where values >0.14 indicate a great effect of the time. The interaction of the time and the group Partial ή^2^ (Time × Group) is analyzed based on the values of Wilk’s lambda. According to the results for this parameter presented in Table 4, it could be noted that the impact of the interaction of the variables time and group are statistically insignificant (*p* < 0.05). Higher and statistically significant values for the indicator Partial ή^2^ (Time × Group) are obtained for the last two factors (F6) Promoting holistic health and (P7) Knowledge and self-awareness of individual application of Brain Break video. This indicates a significant difference between the two groups at pre-test and post-test. The gains from the pre-test and post-test are different in experimental and control groups, as indicated by the significant Time × Group effect. As illustrated in Figure 1, the experimental group gained significantly more than the control group from the pre-test to post-test, resulting with substantially higher scores in the experimental than the control group at post-test.

## 4. Discussion

Different studies have demonstrated the positive relations between the level of physical activity and academic achievements in school children [61,62], the positive effect of physical activity and active break on cognitive functions and brain health [37,38,39], as well as the positive impact of classroom based active breaks in increasing the level of physical activity among children [23,34,35], holistic development [28,63,64] and school behavior [22]. Some of these findings were confirmed in this study as well. 

Initiated by the idea to explore different strategies that can help increasing the level of physical activity in school children supported by new technologies, the implementation of Brain Break^®^ was initiated in Macedonian schools. This propelled the idea for this study, which primary aim was to investigate the effects of implementation of classroom based physical activity breaks on student’s attitudes toward physical activity after three months intervention. Student’s attitudes toward physical activity were evaluated using Macedonian version of Physical activity Scale (APAS). It can be successfully applied for measuring the following seven categories: Self-efficacy in learning with video exercises (F1), Self-confidence in physical fitness (F2). Exercise motivation and enjoyment (F3), Importance of exercise habit for health (F4), Training for personal best and motivating others (F5), Promoting holistic health (F6) and Knowledge and self-awareness of individual application of BRAIN BREAK video (F7). The determined structure of Macedonian APAS is very similar with the structure of Turkish version of the scale, applied in the study of Uzunoz et al. [24]. Out of the seven isolated factors for the Macedonian version, six similar factors were also confirmed in the Turkish study. A more clear structure in our study is noted for the factor named self-efficacy in learning and video exercises. Macedonian version of the scale revealed valid and reliable results for schoolchildren from 3th to 5th grade and it’s recommended for future use with similar sample of examiners. High coefficients of correlation obtained between all seven isolated factors suggest that more frequent use of Brain Break^®^ videos will not have only isolated impact on one developmental aspect, but it will have effects on the development of several aspects including physical fitness, learning experiences through video exercises, striving for personal best achievements, self-awareness of effects of movement habits and their benefits for holistic health and confidence for future individual application of Brain Break^®^ activities. These findings for multiple effects of active breaks in children are also confirmed in the previous studies [22,65,66]. However, it must be highlighted that Brain Breaks video exercises could not replace the regular physical education classes and should not be used as their alternative. Furthermore, the main goal of suggested video exercises is to be used during the classes for other subjects and only as a short active break. Different type of movements incorporated in video exercises available at the platform can be used as a support in the learning process at physical education classes. This type of learning could be realized as inter subject correlation and inter content correlation. In this regard, videos that integrates dance, music, different traditional instruments and traditional costumes could be used as a powerful tool for inter subject correlation. Videos related with different sports, different fundamental movements can be used for inter content correlation and effective strategy for facilitating the learning process of new movements at physical education classes. Used in this manner, Brain Break video exercises could be used both as a method of learning and teaching strategy. This supports the idea of curricular learning at classes and non-curricular learning during short breaks as components of educational process in schools [24]. The effectiveness of application of technology supported brain breaks is highly depended from teacher experience, creativity, personal motivation as well as teacher’s skills to use and implement IT technology in everyday teaching routine [67].

Comparing the results from the experimental and control group in initial measurement, it is evident that they are not equivalent according to the pre-test scores on APAS. In order to determine the reason for this, additional procedures for equalizations and homogenization of both groups should be performed. This fact also felt to be one of the biggest limitations of the study that should be considered in future. Comparing the scores of both groups in the pre-test measurement, numerically, but statistically not significant differences between both groups could be noted. Analyzing the pre-test mean scored in each of the scales, the highest one in both groups was Training for personal best and motivating others (F5), followed by Importance of exercise habit for health (F4), Self-confidence in physical fitness (F2). Exercise motivation and enjoyment (F3), Knowledge and self-awareness of individual application of Brain Break^®^ video (F7), Promoting holistic health (F6) and self-efficacy in learning with video exercises (F1). These results emphasize the motivation of students to engage in Brain Break^®^ video exercises, their awareness of positive effects of these exercises on movement habits, health and overall wellbeing, but students did not understand well the impact that the exercises could have on their learning experience. The same distribution of mean scores is noted in both groups in the final measurement. 

Comparing the post-test mean scores of the experimental and control group, only a small gain from initial to final measurement in each scale is noted for the control group. The highest gain in post-test within the experimental group is noted for the scale “Knowledge and self-awareness of individual application of Brain Break^®^ video, expressing the gain confidence to apply Brain Break^®^ video independently. This is very encouraging information if we analyze it from the point of future use of technology supported video exercises and possibility to practice Brain Break^®^ video activities in school and at home as well. To supplement our findings, Caldwell & Ratliffe (2014) [68] reported that a very brief message on the benefits of regular exercise along with the exercise clips would make this session more meaningful to the children in terms of knowledge and self-awareness.

The second highest gain in mean scores in the experimental group is noted for the scale self-efficacy and learning with video exercises, which is very meaningful and important from the aspect of integration of Brain Break^®^ activities into everyday school routine. The finding is encouraging as previous studies described self-efficacy or physical activity confidence as being one of the most dominant correlates of active life-long engagement in physical activity [69,70]. From the aspect of learning with video exercises, it is important to highlight that technology supported video exercises could be successfully used, not just as a tool for active break and increase of physical activity level, but also as a meaningful tool for establishing cross-subject relations, integrations and holistic learning. Applied Brain break activities intervention combines video exercises with specific academic knowledge in the areas of language, music, art, culture, composition, mathematics and environmental protection as well as with health related–related knowledge (healthy lifestyle, healthy diet). Similar results related with effects on Brain Break intervention on Self-efficiency and learning are also obtained in the study of Sacli et al. [24]. The authors emphasize the benefits of using Brain Break^®^ video activity in general education, positioning “the education in schools as a continuous process interchanging curricular education during classes and non-curricular education during short active breaks” [24] (p. 96). Several study reports support the positive effects of physical activity and active break on children learning and academic performance [4,34,66,71] and behavior in the class [22]. The use of classroom based physical activity, as tool for integrating teaching contents from different school subjects is a very important goal of Brain Break^®^ video considering the importance of this process for children development [63,72]. Speaking about effects on students learning, reports from numerous studies confirm the positive effect of physical activity and active break on students attention and on-class behavior [40,41,65], cognitive functions and brain health [4,36,37,38]. 

Higher and statistically significant values for the indicator Partial ή^2^ (Time × Group) obtained for the last two factors (F6) Promoting holistic health and (P7) Knowledge and self-awareness of individual application of Brain Break^®^ video could be explained with the effects of some other factors that should be additionally investigated. Additional statistical analyses of these parameters in future could provide more concrete information about the impact of other external factors, which indicate such condition. This could be considered to be one of the limitations of the current study. 

This study confirmed the effect of the application of Brain Break^®^ video exercises on children’s positive attitudes to physical activity, motivation for physical activity, internalization of movement habits as personal good, something worth to work on, but it also emphasizes the effects related to the learning process, socialization, mutual interaction and positive emotions. In the researchers’ opinion, the effectiveness of this process is highly correlated with the information, motivation and knowledge of the teacher how to use Brain Break^®^ video exercises, not just as 3–5 active breaks, but also as learning and teaching strategy. This opinion and implication has been supported by the previous investigations that the influence of committed classroom teachers has a great potential to enhance the daily activity patterns of children [73,74]. In this regard, analyzing the contents of the applied Brain Break^®^ video exercises, a variety of movements, dances, music, costumes, steps, environments etc. could be noted. Using all these different aspects in certain educational situations could provide a possibility to include video exercises in the teaching and learning material, having in mind that these physical activity breaks are usually non-competitive and individually based in nature. Considering the various benefits of using Brain Break^®^ videos, one important question arises and it is related to the manner of its use. Namely, issues related to the selection of video, period of application, possibility for cross-subject correlations mainly depend on the teacher. This means that teachers should be well prepared and informed about all possibilities and benefits from the use of Brain Break^®^ videos. In this regard, positive attitudes of teachers toward the use of technology, the manners and frequency of its application as well as the preparedness to get familiar with the advantages of technology and possibility to use it effectively in practice could be underlined as essential for the successful application of technology in the teaching process [67].

Another point arising from the analyses of contents of Brain Break^®^ videos suggested in the Video Library is the variety of movements with different complexity, structure and intensity. Many of these physical activity movements, practiced and repeated each day will not just have effect on cognitive functions of the children [75], but they can also have certain impact on some motor abilities and skills in children, mainly in the segment of coordination, agility and rhythmic structures [76,77]. Therefore, one of the suggestions for future actions is designing a study that will investigate not just changes in attitudes, but also changes in motor achievements and motor abilities of children. 

The association of the findings of this study to the future application in global holistic education can be implied from the partnership of The Foundation for Global Community Health (GCH) (with HOPSports Brain Breaks^®^ Physical Activity Solutions with the United Nations Global Sustainability Index Institute (UNGSII) on 5 June 2017. The goal of this co-operation is to promote the UN Global Sustainable Development Goals (SDGs), educational programs for children from all 193 countries that signed the United Nation 17 global goals by building SDG Labs in schools around the globe [78]. 

## 5. Conclusions

The results of this study confirm the positive effects of three months intervention of classroom based HOP Sports Brain Breaks^®^ Physical Activity Solutions mainly in improvement of Children’s’ knowledge and Self-awareness for individual application of Brain Break activities and Promoting the holistic health. Frequent use of classroom based Brain Break^®^ video exercises demonstrated positive changes in children attitude for motivation for physical activity as well as positive impact on several developmental aspects emphasizing the holistic approach in learning and teaching,. In this regard, we recommend implementation of Brain Break^®^ activities in everyday school routine not just as a tool for active break and motivation for physical activity, but also as a meaningful tool for establishing cross-subject relations, integration, holistic learning and development. 

The process of implementation of Brain Break^®^ physical activities is easy to be performed in school setting. Yet, the effectiveness of this process is highly related to the work and attitudes of teachers. In this regard, positive attitudes of teachers toward the use of technology, manners and frequency of its application as well as the preparedness to get familiar with the advantages of technology and the possibility to use it effectively in practice could be underlined as essential for the successful application of technology in the teaching process. Therefore, future efforts in promoting HOPSports Brain Breaks^®^ Physical Activity Solutions should be pointed not only to the promotion of benefits and effects that these solutions have on children, but also on the promotion of gains for the teaching process and effectiveness of teachers’ work. In this regard, future investigations should be addressed to the richness of learning based on students’ experiences, improvements in students’ behavior and movement habits. 

We strongly support the idea of creating video exercises by both teachers and children. We consider that such participation will support the creativity of both teachers and children and will strengthen the learning and teaching processes.

## Figures and Tables

**Table 1:** General characteristics (mean, standard deviation and frequency) of the sample of the participants.

Variables	Total *n* = 238		Control Group *n* = 131 (46%)		Experimental Group *n* = 152 (54%)	
Age (years)	9.21	0.97	9.18	1.13	9.24	0.82
Body height (m)	1.39	0.16	1.37	0.21	1.41	0.10
Body weight (kg)	36.70	8.84	35.97	9.89	37.32	7.81
Gender						
Male	155	54.8%	69	52.5%	86	52.7%
Female	128	45.2%	62	47.3%	66	47.3%
BMI categories						
Under weight	16	5.70%	13	9.90%	3	2.00%
Normal weight	159	56.20%	72	55.00%	87	57.20%
Over weight	61	21.60%	24	18.30%	37	24.30%
Obese	46	16.30%	21	16.00%	25	16.40%
Grade level						
Grade 3	97	34.30%	45	34.40%	52	34.20%
Grade 4	105	37.10%	50	38.20%	55	36.20%
Grade 5	81	28.60%	36	27.50%	45	29.60%

**Table 2:** Factor structure of APAS.

Items	Items name	Factors						
		1	2	3	4	5	6	7
Self-efficacy in learning with video exercises (F1)								
P3d	I learned about mathematics through video exercise.	0.785						
P3f	I learned about writing through video exercise.	0.782						
P3c	I learned about art through video exercise.	0.771						
P3h	I learned about healthy lifestyle from video exercise.	0.714						
P3g	I learned about composition through video exercise.	0.708						
P3k	I learned about environmental protection from video exercise.	0.700						
P3j	I learned about hygiene from video exercise.	0.694						
P3i	I learned about healthy diet from video exercise.	0.676						
P3b	I learned about music through video exercise.	0.671						
P3e	I learned about language through video exercise.	0.658						
P3a	I learned about culture through video exercise.	0.568						
P1j	Being physically active helps to improve my schoolwork.	0.501						
Self-confidence on physical fitness (F2)								
P6g	I am confident with my hand-eye coordination.		0.763					
P6d	I am confident with my agility.		0.745					
P6b	I am confident with my endurance.		0.709					
P6h	I am confident in doing physical activity elegantly.		0.706					
P6c	I am confident with my balance.		0.703					
P6a	I am confident with my strength.		0.700					
P6f	I am confident with my rhythm.		0.627					
P6e	I am confident with my flexibility.		0.601					
Enjoyment and exercise motivation (F3)								
P5k	I think my classmates enjoy doing physical activity.			0.685				
P5l	I think other children enjoy doing physical activity.			0.663				
P5n	I think my parents/guardians enjoy physical activity.			0.614				
P5d	I achieve my physical activity goals even if I am tired.			0.594				
P5j	I improve on my school work after physical activity.			0.576				
P5b	I look forward to doing physical activity.			0.575				
P5a	I think physical activity is fun.			0.567				
P5f	I feel better after physical activity.			0.551				
P5g	I feel stronger after physical activity.			0.503				
P5c	I enjoy doing physical activity with my classmates.			0.489				
P5i	I think better after physical activity.			0.465				
P5h	I feel more confident after physical activity.			0.411				
Importance of exercise habit for health (F4)								
P2a	It is important to spend time to be physically active				0.762			
P2c	It is important to be physically active for my health.				0.656			
P2b	It is important to form a habit of being physically active.				0.635			
P2e	Even if I have a lot of work to do, I still keep being physically active.				0.585			
P1h	Being physically active helps to give me good health.				0.545			
P1a	Being physically active helps to make me fit.				0.489			
P1i	Being physically active helps to improve my sleep.				0.434			
Training for personal best and motivating others (F5)								
P7e	I seek to explore my best potential in physical activity.					0.689		
P7c	I keep striving for breakthroughs in physical activity.					0.686		
P7d	I do not compare with others but just do my personal best in physical activity.					0.679		
P7b	My target is to go beyond what I have achieved in physical activity.					0.641		
P7a	I try my best to engage in physical activity					0.629		
P5e	I persuade my friends to join me in doing physical activity.					0.411		
Promoting holistic health (F6)								
P1c	Being physically active helps to reduce my anxiety.						0.654	
P1e	Being physically active helps to enhance my self-concept.						0.633	
P1d	Being physically active helps to improve my analytic skills.						0.590	
P1f	Being physically active helps to give me new experience every time.						0.534	
P1g	Being physically active helps to give me more willpower.						0.519	
P1b	Being physically active helps to refresh my thinking.						0.433	
Knowledge and self-awareness for individual application of BB video (F7)								
P4b	I know how to do physical activity if there is a video exercise to follow							0.657
P4d	I know which my favorite physical activity is in video exercises.							0.624
P4a	I know how to choose physical activity in video exercises that suits me.							0.559
P4c	I can follow physical activity in video exercises with minimal mistakes even without a teacher.							0.513
	Eigenvalues	16.17	5.94	2.51	2.21	2.08	1.63	1.38
	Percentage of explained variance	28.36	10.41	4.41	3.88	3.65	2.85	2.42
	Percentage of total explained variance	28.36	38.7	43.19	47.07	50.71	53.57	55.98

**Table 3:** Correlation between the factors.

	PFAK1	PFAK2	PFAK3	PFAK4	PFAK5	PFAK6	PFAK7
PFAK1	1.000						
PFAK2	0.209 **	1.000					
PFAK3	0.351 **	0.522 **	1.000				
PFAK4	0.154 **	0.400 **	0.438 **	1.000			
PFAK5	0.155 **	0.555 **	0.611 **	0.499 **	1.000		
PFAK6	0.457 **	0.351 **	0.440 **	0.413 **	0.350 **	1.000	
PFAK7	0.417 **	0.359 **	0.328 **	0.302 **	0.372 **	0.390 **	1.000

** *p* < 0.01

**Table 4:** Pre-test & post-test mean scores, differences between time points and between experimental and control group.

Variables on Physical Activity	Group	Pretest M (SD)	Posttest M (SD)	Partial *ή*^2^ (Time)	Partial *ή*^2^ (Time × Group)
Self-efficacy in learning with video exercises (F1)	Experimental	2.88 (0.87)	3.07 (0.87)	0.017 *	0.009
	Control	2.60 (0.76)	2.63 (0.75)		
Self-confidence on physical fitness (F2)	Experimental	3.47 (0.59)	3.56 (0.51)	0.028 *	0.000
	Control	3.16 (0.63)	3.28 (0.65)		
Exercise motivation and enjoyment (F3)	Experimental	3.48 (0.56)	3.58 (0.49)	0.013 *	0.002
	Control	3.17 (0.61)	3.22 (0.62)		
Importance of exercise habit for health (F4)	Experimental	3.58 (0.58)	3.70 (0.46)	0.016 *	0.003
	Control	3.30 (0.56)	3.34 (0.62)		
Training for personal best and motivating others (F5)	Experimental	3.62 (0.51)	3.74 (0.41)	0.21 *	0.001
	Control	3.39 (0.59)	3.46 (0.58)		
Promoting holistic health (F6)	Experimental	3.12 (0.77)	3.38 (0.51)	0.25 *	0.041 *
	Control	2.93 (0.58)	2.90 (0.61)		
Knowledge and self-awareness for individual application of Brain Break video (F7)	Experimental	3.16 (0.72)	3.62 (0.50)	0.66 *	0.102 *
	Control	2.83 (0.68)	2.79 (0.73)		

Note: * *p* < 0.01.
